# Case Report: Molecular Diagnosis of Fungal Keratitis Associated With Contact Lenses Caused by *Fusarium solani*

**DOI:** 10.3389/fmed.2021.579516

**Published:** 2021-03-24

**Authors:** Laura Trovato, Antonio Marino, Giovanni Pizzo, Salvatore Oliveri

**Affiliations:** ^1^Laboratory Analysis Unit, Azienda Ospedaliero Universitaria Policlinico “G. Rodolico-San Marco”, Catania, Italy; ^2^Department of Biomedical and Biotechnological Sciences, University of Catania, Catania, Italy; ^3^Ophthalmology Unit, Azienda di Rilievo Nazionale e di Alta Specializzazione Garibaldi, Catania, Italy

**Keywords:** *Fungal keratitis*, *Fusarium solani*, contact lenses, diagnosis, PCR

## Abstract

*Fusarium* is a filamentous fungus commonly found in the environment and is the major cause of fungal keratitis. We report a case of keratomycosis caused by *Fusarium solani* in a patient using disposable soft contact lenses. A delay in diagnosis led to the initiation of an empirical antifungal treatment with the subsequent deterioration of the patient's clinical condition. The use of the real-time quantitative PCR assay confirmed keratitis from *F. solani* providing a result in <48 h and therefore giving the possibility of quickly starting targeted antifungal therapy. The patient had an improvement in eye condition after the diagnosis of keratitis by *F. solani* and the rapid change to targeted antifungal treatment. For the rapid identification of corneal fungal pathogens, we believe that PCR may be added for the diagnosis of mycotic keratitis pending the isolation in culture that is necessary for *in vitro* susceptibility testing.

## Introduction

Fungal keratitis (FK) is a potentially sight-threatening fungal infection of the cornea and has been reported to account for about half of all microbial keratitis cases requiring therapeutic penetrating keratoplasty (1, 2). FK develops rapidly and can lead to corneal ulcers and vision loss; therefore, early diagnosis and prompt treatment are essential to prevent long-term complications. It is typically caused by *Aspergillus* species, *Candida* species, and several species of the genus *Fusarium*, mostly *Fusarium solani* that is the most virulent, associated with the ability to generate resistance to many antifungal agents (3–7). Trauma to the cornea is the most common risk factor for FK, followed by topical corticosteroid use, ocular surface disease, contact lens use, and systemic immunosuppression (8–10). In all patients with a suspicion of keratomycosis, a timely diagnosis is among the most important factors because an early diagnosed episode of keratomycosis could favor a good prognosis (11).

## Case Report

A 25-year-old female, who had been using disposable soft contact lens for several years, was assessed for a sensation of pain in her left eye. A sty was diagnosed, and medical therapy with tobramycin eye drops was prescribed.

**Figure 1:**
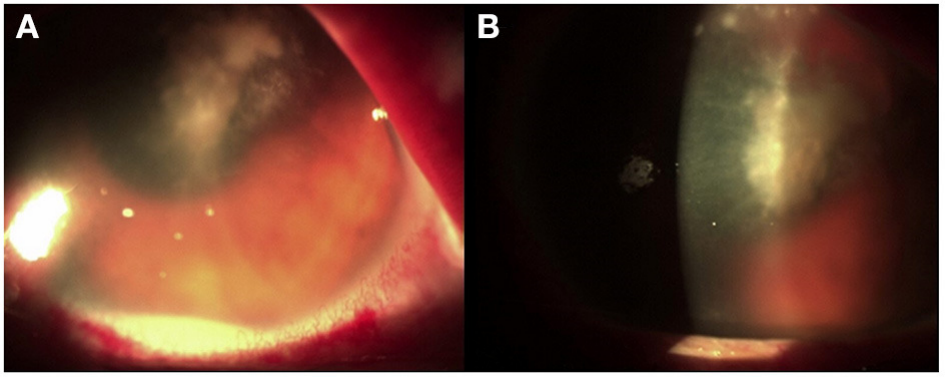
**(A)** Corneal abscess with fibrinous branches. **(B)** Collection of purulent inflammatory exudate.

**Figure 2:**
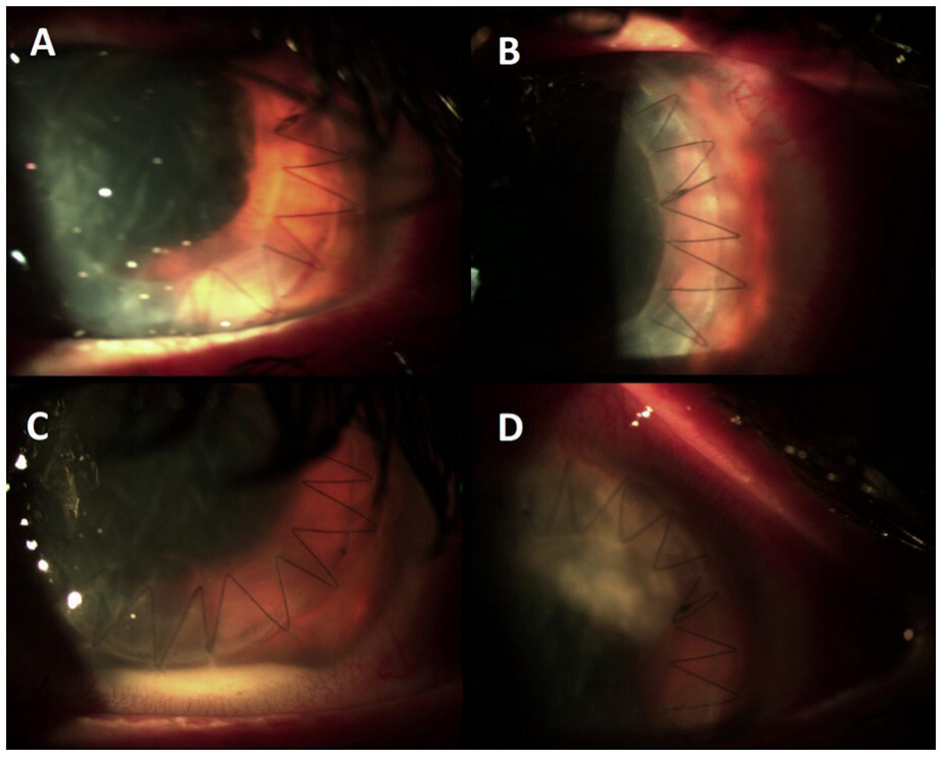
**(A,B)** Condition after corneal transplant at the time of discharge. **(C)** Appearance of hypopyon and abscess **(D)** at the time of the second hospitalization.

After 10 days, despite topical antibiotic therapy for the persistence of pain, the patient referred a reduction of visual acuity to 6/15, and because of the appearance of diffuse edema at the epithelial level, *Acanthamoeba* keratitis was suspected; a swab was taken and analyzed together with her contact lenses for a microbiological examination. Other than therapy with tobramycin drops every 2 h, Polihexanide 0.02% (PHMB) eye drops were also administered (2 drops × 6 v/die). The patient's condition subsequently deteriorated rapidly, and 3 days later, she was hospitalized. On local examination, corneal abscess with a collection of purulent inflammatory exudate of ~1 mm in size was observed (Figure 1). No view of the lens or fundus was possible, and vision was impaired. Routine blood tests were normal, and the search for *Acanthamoeba* from the swab and contact lenses was negative. Therapy with vancomycin intravenous 1 g daily and ceftazidime 1 g every 12 h daily was started. Atropine drops 3 per day, levofloxacin and tobramycin drops every 2 h, and PHMB (2 drops × 6 v/die) were also administered. Corneal scraping was performed 4 days later and sent to the Clinical Pathology Service that reported the presence of yeast cells on Gram stain, and that there was a fungal culture in progress. Considering the suspicion of probable *Candida* infection, vancomycin was changed to topical fluconazole (200 mg/100 ml) 1 drop every hour, and caspofungin intravenous (50 mg/die) was also administered, whereas the PHMB eye drops and tobramycin drops were stopped. After 6 days, the anterior chamber was washed with fluconazole (200 mg/100 ml), and fibrinous tissue was taken and reported as fibrin-leukocyte exudate. After a slight improvement in symptoms, the patient's condition worsened, and after a further anterior chamber washing with fluconazole, a corneal transplant was performed with removal of the membrane that covers the corneal iris angle and the crystalline lens, followed by intraocular lens (IOL) implantation. The patient was discharged from the hospital after 10 days. On the 26th day after the transplant, she was hospitalized again due to a worsening of her clinical condition (Figure 2).

After 2 days, a new corneal transplant was performed. The explanted corneal flap and the purulent material of the anterior chamber were sent to the Mycology Laboratory of the University Hospital of Catania, Sicily, Italy. For the mycological examination, conventional and molecular diagnostic methods by microscopic, fungal culture in Sabouraud Dextrose Agar (SDA) medium supplemented with 1% chloramphenicol and gentamycin and real-time quantitative PCR (qPCR) assay for the detection of *Fusarium* and *Aspergillus* were carried out.

The qPCR assay was used for the detection of *Fusarium* spp. using the primers and probes as described by Koo et al. (12), whereas the AsperGenius® multiplex PCR (PathoNostics, Maastricht, Netherlands) was used to detect the most clinically relevant *Aspergillus* species. DNA was extracted by using the GenoXtract instrument (Hain Lifescience, Nehren, Germany) following the manufacturer's instructions. qPCR was performed adding 5 μl of DNA extract to the PCR mix, and a Rotor-Gene Q (Qiagen) was used for amplification and melting curve analysis. A positive control (*Fusarium falciforme* ATCC® MYA3636™) and a negative template control (NTC) were included in each PCR run. The AsperGenius® multiplex PCR was performed according to the manufacturer's instructions.

**Figure 3:**
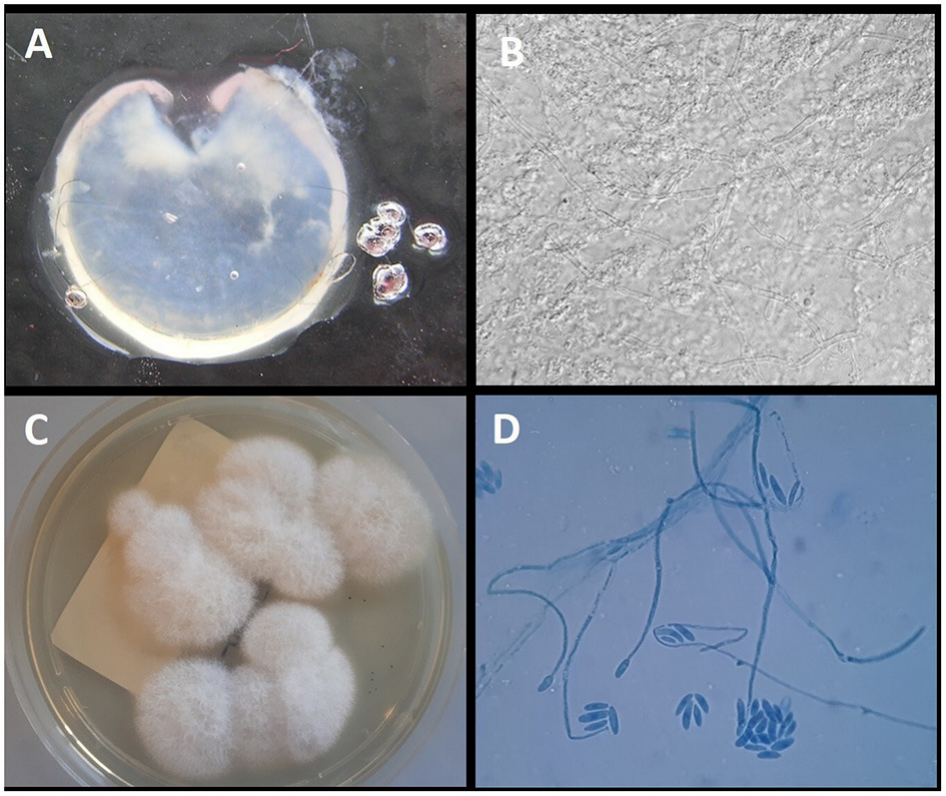
**(A)** Explanted corneal flap. **(B)** Direct examination of the corneal flap with 15% KOH (original magnification ×40); **(C)** growth on Sabouraud Dextrose Agar with gentamycin and chloramphenicol after 7 days of incubation at 32°C; **(D)** Microscopic structure of the colony showing long monophialidic conidiogenous cells and numerous microconidia of *F. solani*.

The direct microscopic examination with 15% potassium hydroxide (KOH) showed several hyaline septate hyphae. *Fusarium* spp. was detected by qPCR, whereas *Aspergillus* DNA was negative. After 4 days of incubation at 30°C, the colonies were clearly visible on SDA medium. The colonies were wooly, cottony, flat, and white. Identification of the isolate was performed by standard phenotypic methods based on the macroscopic and microscopic morphological studies. In particular, the microscopic morphological study showed long monophialidic conidiogenous cells, moderately curved macroconidia and cylindrical to oval microconidia with thick walls, and single-celled to two-celled. The pathogen was identified as *F. solani* complex. The results of the mycological examinations are shown in Figure 3.

**Figure 4:**
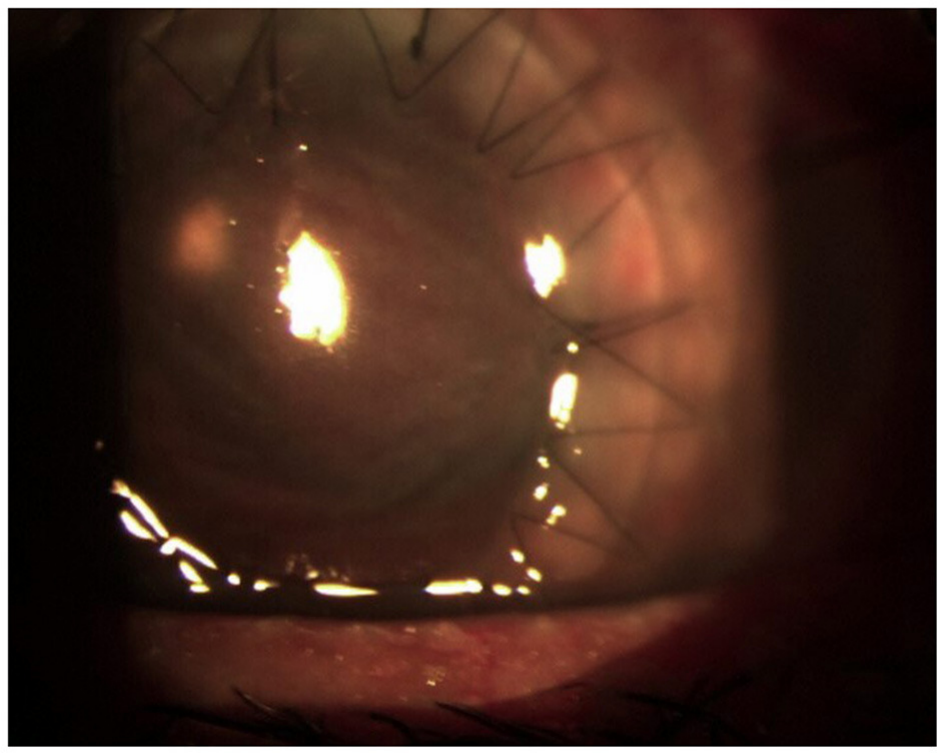
Condition after the second transplant.

Matrix-assisted laser desorption ionization time of flight mass spectrometry (MALDI-TOF MS) on a Microflex LT (Bruker Daltonics, Bremen, Germany) platform after ethanol–formic acid extraction identified the isolate as *F. solani* (score: 1.646). Susceptibility to fluconazole, itraconazole, voriconazole, posaconazole, caspofungin, anidulafungin, micafungin, flucytosine, and amphotericin B was evaluated. Minimum inhibitory concentration (MIC) values of >256 μg/ml for fluconazole, >16 μg/ml for itraconazole, 0.256 μg/ml for voriconazole, >8 μg/ml for posaconazole, >8 μg/ml for caspofungin, >8 μg/ml for anidulafungin, >8 μg/ml for micafungin, >64 μg/ml for flucytosine, and 1 μg/ml for amphotericin B were obtained. The patient was put on topical voriconazole eye drops every h and voriconazole 200 mg i.v. twice a day, and there was an almost immediate improvement of her eye condition (Figure 4). The systemic therapy with voriconazole 200 mg per os twice a day was continued for at least 3 months postoperatively.

At 3 months, despite the control of her fungal infection, the cornea is opaque due to a fibrinous reaction that also affects the IOL. After controlling the infection, the visual function can probably be restored with a new cornea transplant and replacement of the IOL.

## Discussion

We report a case of keratomycosis caused by *F. solani* in a patient who had been wearing disposable soft contact lens for several years, often also used overnight. Outbreaks of *Fusarium* keratitis associated with the use of specific contact lens solutions as well as the ability of *Fusarium* to penetrate soft contact lenses forming biofilm have been described (13). Keratomycosis caused by *Fusarium* is important among the clinical conditions responsible because of ocular morbidity and blindness. It is a clinical challenge due to its slow pathological process, characteristics similar to other microbial keratitis, and possible complications. Therefore, a timely diagnosis and adequate therapy are the most important factors that favor a good prognosis. In our case, an *Acanthamoeba* keratitis was initially suspected but was excluded after the negative results of the tests carried out on the swab and contact lenses. Later, a probable *Candida* keratitis was suspected based on the presence of yeast cells on Gram stain after corneal scraping.

This determined the start of an empirical treatment with topical fluconazole and intravenous caspofungin; however, there was a subsequent deterioration of her clinical condition. Considering the worsening clinical condition of the patient, notwithstanding antifungal therapy, we excluded *Candida* as a possible etiological agent of keratitis because hypha formation is an essential step in the pathogenesis of *Candida albicans* in keratitis (14) and also in light of it not even being isolated in culture. Probably, in this initial phase, had the corneal scraping been sent to a specialized laboratory equipped for molecular and conventional mycologic diagnoses, a correct diagnosis could have been made, and a correct antifungal treatment could have been started without the necessity of a second transplant.

In fact, only at the second transplant, on the explanted corneal flap, was FK due to *F. solani* confirmed and treatment with topical and intravenous voriconazole started that resulted in an improvement of the condition of her eye.

The gold standard of laboratory diagnosis of FK includes the microscopic examination by histopathology or the KOH preparation of corneal scraping and a fungal culture. Generally, the sensitivity of the KOH examination may be negatively affected by the insufficient amount of scraping material, the small size of the corneal ulcer, and the lack of experience of the microscope observer. Moreover, the Gram stain, commonly used in clinical laboratories, has a significantly lower sensitivity than the KOH examination (15). The cultures, which are widely used in clinical laboratories, remain negative in several positive microscopy cases, as well as being associated with a long turnaround time.

In our case, FK was confirmed by microscopic examination in KOH, isolation in culture, and PCR even if the use of the qPCR assay identified *Fusarium* sp. keratitis in <48 h. PCR, as in other clinical settings (16), proved to be an effective, rapid method for the diagnosis of FK and was more sensitive than microscopy and culture methods; therefore, we believe that PCR should be added as a screening diagnosis test when an early mycotic keratitis is suspected (17).

However, there are problems related to the diagnosis and the management of *Fusarium* keratitis; considering that *Fusarium* species exhibit broad resistance to the spectrum of antifungals currently available, the isolation in culture is important to carry out an *in vitro* susceptibility. Data on their *in vitro* susceptibility to various antifungal agents indicate variable susceptibility to amphotericin B and extended-spectrum triazoles, such as itraconazole, voriconazole, isavuconazole, and posaconazole (18). The fungal sample that was isolated in our case showed susceptibility to amphotericin B and voriconazole with MIC values of 1 and 0.256 μg/ml, respectively, whereas resistance was demonstrated against all the other tested antifungals.

In conclusion, for the rapid identification of corneal fungal pathogens, we believe that PCR should be added for the diagnosis of mycotic keratitis pending the isolation in culture necessary for *in vitro* susceptibility tests. However, the diagnostic challenge of FK lies in the use of laboratories with expertise in medical mycology so as to assure an appropriate diagnostic course correlated to the presentation of the clinical picture.

## Data Availability Statement

The original contributions generated for the study are included in the article/supplementary material, further inquiries can be directed to the corresponding author/s.

## Ethics Statement

Written informed consent was obtained from the individual(s) for the publication of any potentially identifiable images or data included in this article.

## Author Contributions

LT and SO performed the conventional and molecular diagnoses and wrote the manuscript. AM and GP performed the image acquisition and contributed the clinical details. All authors read and approved the manuscript.

## Conflict of Interest

The authors declare that the research was conducted in the absence of any commercial or financial relationships that could be construed as a potential conflict of interest.
